# Chlorophyll Oxidative Metabolism During the Phototrophic and Heterotrophic Growth of *Scenedesmus obliquus*

**DOI:** 10.3390/antiox8120600

**Published:** 2019-11-29

**Authors:** Mariana Manzoni Maroneze, Leila Queiroz Zepka, Eduardo Jacob Lopes, Antonio Pérez-Gálvez, María Roca

**Affiliations:** 1 Department of Food Science and Technology, Federal University of Santa Maria (UFSM), 97105-900 Santa Maria, Brazil; mariana_maroneze@hotmail.com (M.M.M.); lqz@pq.cnpq.br (L.Q.Z.); jacoblopes@pq.cnpq.br (E.J.L.); 2 Food Phytochemistry Department, Instituto de la Grasa, Consejo Superior de Investigaciones Científicas (CSIC), University Campus, Building 46, Carretera de Utrera km. 1, 41013 Sevilla, Spain; aperez@ig.csic.es

**Keywords:** phototrophic, heterotrophic, *Scenedesmus*, chlorophylls, carotenoids, hydroxy-chlorophyll, oxidative metabolism, ROS, lactone-chlorophyll, photoacclimation

## Abstract

Different cultivation strategies have been developed with the aim of increasing the production rate of microalgal pigments. Specifically, biotechnological approaches are designed to increase antioxidant metabolites as chlorophyll and carotenoids. However, although significant advances have been built up, available information regarding both the chlorophyll metabolism and their oxidative reactions in photobioreactors is scarce. To unravel such processes, the detailed chlorophyll and carotenoid fraction of *Scenedesmus obliquus* has been studied by HPLC-ESI/APCI-hrTOF-MS from phototrophic and heterotrophic cultures. *Scenedesmus* is provided with a controlled strategy of interconversion between chlorophyll *a* and *b* to avoid the formation of reactive oxygen species (ROS) at high irradiances in addition to the photoacclimation of carotenoids. Indeed, precise kinetics of 13^2^-hydroxy- and 15^1^-hydroxy-lactone chlorophyll metabolites shows the existence of a chlorophyll oxidative metabolism as a tool to manage the excess of energy at high light conditions. Unexpectedly, the oxidation under phototrophy favored chlorophyll *b* metabolites over the chlorophyll *a* series, while the heterotrophic conditions exclusively induced the formation of 13*^2^*-hydroxy-chlorophyll *a*. In parallel, during the first 48 h of growth in the dark, the chlorophyll fraction maintained a promising steady state. Although future studies are required to resolve the biochemical reactions implied in the chlorophyll oxidative metabolism, the present results agree with phytoplankton metabolism.

## 1. Introduction

Chlorophyll and carotenoids are challenging compounds in microbial biotechnology that find several applications in the food industry. The present food market trend is towards more natural ingredients, and colorants are not an exception [1]. Hence, artificial food colorants have been associated with health problems and consequently new sources of natural colorants are under investigation. Although natural food colorants have been traditionally extracted from fruits and vegetables sources, microalgae are currently a promising natural resource. Several advantages as the fast growth, the high pigment concentration, and the physiologically plasticity, make microalgae the new objective of biotechnological companies for pigment production. Among them, *Scenedesmus obliquus* stands out as source of pigments in food and cosmetics as well as is considered for human consumption [2,3]. In addition, chlorophyll and carotenoids exert beneficial health properties for human beings that increase their value as functional ingredients [4]. Specifically, both groups of pigments have shown to develop antioxidant activities. The antioxidant behavior of chlorophylls is highly dependent of the type of chlorophyll derivative, with significant antioxidative performances among metabolites [5]. The porphyrin structure, the central magnesium, and the functional group at C7 seem to be determinants for the antioxidant activity [6,7,8,9]. In the same line, carotenoids are highly appreciated by their antioxidant properties [10]. Consequently, the production of chlorophyll and carotenoids is one of the most successful applications of microalgal biotechnology [11], although the improvement of the feasibility of their commercial production through better cultivation strategies is still the main goal.

Photo-autotrophy is the classical culture system to grow microalgae, where the energy source comes from the sunlight and the carbon source from the atmospheric CO_2_. Different light regimes (intensity, photoperiod, and wavelengths) generate different chlorophyll and carotenoid patterns. It is generally assumed that sub saturating light intensities induce higher chlorophyll synthesis, while high light irradiation reduces the chlorophyll content [12]. On the contrary, specific microalgae (*Haematococcus pluviales, C. zofingiensis*) enhance the production of secondary carotenoids when grown with high light intensities [13].

Open and closed photobioreactors present several disadvantages, such as the presence of contaminants, the need of robust species, or the requirement of vigorous mixing [13]. Consequently, growing microalgae in heterotrophic conditions in conventional bioreactors is at present an attractive and economical option. An additional advantage is that green algae can synthesize chlorophylls in dark conditions unlike angiosperms. This is possible thanks to the presence of a light-independent POR (protochlorophyllide *a* oxidoreductase) enzymatic process, one of the key enzymes in the chlorophyll biosynthetic pathway [14]. However, only a few microalgal species have been shown to grow in the dark so far, because the capacity of sugar utilization is not a universal strategy. In this sense, a constitutive glucose transport and utilization system has been reported for *Scenedesmus obliquus* growth [15]. In fact, different new strategies are continuously developing. Cyclic autotrophic/heterotrophic cultivation, where organic carbon is added during light or during the dark phase has been studied to optimize the production of chlorophylls and carotenoids [16]. The strategy of cultivation in two stages has been explored as an alternative to avoid the division between cell growth and the production of secondary metabolites [17]. The first phase is dedicated to obtaining the maximum biomass production, followed by a stressed second phase to increase the accumulation of lipid-derived compounds. In fact, such strategy (two-stage heterotrophy/photoinduction) has been successfully applied in *Scenedesmus* reaching values of lutein productivity 1.6 times higher than in autotrophic conditions [18]. With the same aim, that is, to enhance the lutein productivity, the conditions during mixotrophic cultures of *S. obliquus* have been optimized, determining the best operating parameters for photoperiod, source of light, nutrients and batch system [11]. Different conditions have been established to modulate, improve, or control the chlorophyll content in different *Scenedesmus* sp. [19,20]. Light conditions, stirring, depletion of nutrients, open fields or bioreactors, and carbon source are among the most explored variables [12]. *Scenedesmus* seems to have a lower sensitivity to photoinhibition and a higher capacity to adapt to high irradiance conditions by increasing its photosynthetic capacity, in comparison with other species such as *Chlorella* [21]. However, the following step is to analyze the chlorophyll profile in detail, to decipher the responsible mechanism(s) for the synthesis and degradation of chlorophylls in response to modifying parameters.

**Figure 1:**
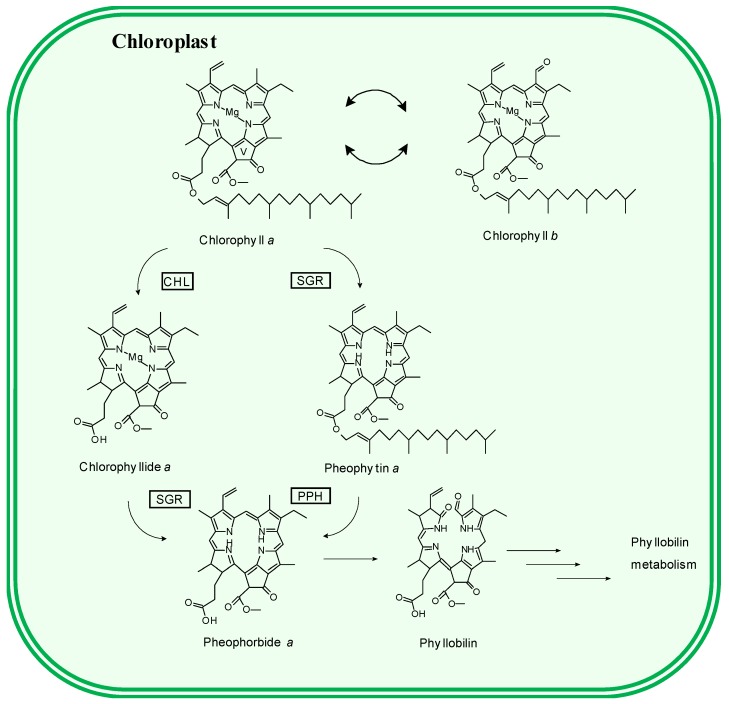
Chlorophyll degradation pathway. CHL: chlorophyllase, SGR: stay-green, PPH: pheophytinase.

**Figure 2:**
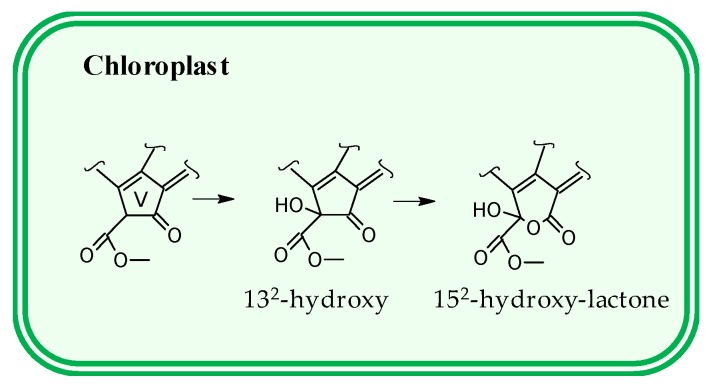
Oxidative chlorophyll reactions, following previous proposal [31]. The structures correspond only with the V ring (or isocyclic ring) of the chlorophyll molecule. The wavy line means the rest of the chlorophyll structure (see Figure 1). The oxidative reactions can be performed over diverse chlorophyll compounds (see the tables).

The biochemical reactions implied during the chlorophyll degradation pathway have been unraveled in higher plants [22], but in green microalgae only a few steps have been discovered [23,24]. Thus, the initial catabolic steps of chlorophyll *a* are the de-esterification of phytol (Figure 1) and the loss of the central magnesium to finally yield pheophorbide *a*. For these consecutive reactions, two plausible alternative routes have been proposed [22], which are potentially catalyzed by different enzymes. The last proposal has been to postulate that both enzymatic systems can operate simultaneously, although at different functional levels [25]. Next, the macrocycle of the pheophorbide *a* intermediate is oxygenolytically open, yielding a sequence of linear chlorophyll catabolites denominated phyllobilins (Figure 1). Regardless, it has been established that knowledge level of chlorophyll degradation in microalgae is at present in a preliminary stage. Specifically, as it was regarding that of the higher plants 30 years ago. A very close relationship has been always suggested between the chlorophyll catabolic pathway in chlorophytes and in higher plants [26], which is not unlikely assuming the phylogenetic relationship between both taxonomic groups. In fact, open chlorophyll catabolites with similar structures to those of phyllobilins have been identified in *Chlorella* [26,27] or in *Desmodesmus subpicatus* [28]. In parallel, chlorophylls are subjected to an oxidative metabolism [29,30]. At present, two reactions have been identified. Hence, the chlorophyll skeleton can be oxidized at C13^2^ to form 13^2^-hydroxy-compounds and secondly, while the isocyclic ring can further react to form a lactone group, that is, the 15^2^-hydroxy-lactone chlorophyll derivatives [31] (Figure 2). Their formation could arise from different pathways, enzymatic [30] or by an increase in oxygen reactive species, and even they can also be produced in dark anoxic conditions [32]. Regardless, the presence of hydroxy-chlorophylls has been related with conditions of high environmental presence of peroxide species, as the former are the main products of chlorophyll *a* in presence of the latter [33]. Therefore, hydroxy-chlorophylls are related to the response to oxidative stress. Physiologically, they have been associated with senescence [34], virus infection [35], and even cell death [36]. In any case, hydroxy-chlorophylls are common chlorophyll metabolites found in phytoplankton species in their natural environments [36,37]. For some authors, oxidative chlorophylls are the origin of “petrochlorophylls”, as they have been identified in numerous phytoplankton sediments [38]. However, to the best of our knowledge, chlorophyll oxidation has not been analyzed in relation to microalgae cell culture.

As stated before [39], a deep knowledge of their metabolic pathways is necessary to select the best cultivation conditions to improve the microalgal pigment production. Although significant advances have been developed to maximize the total chlorophyll and carotenoid content in some microalgae species, it is necessary to understand the individual behavior within the heterogenous pigment profile. It is necessary not only to consider the total pigment content, but also to determine which metabolites are producing or degrading. The aim of this study was to analyze in detail the chlorophyll and carotenoid metabolism during the phototrophic and heterotrophic cultivation of *Scenedesmus* with special emphasis in the oxidative reactions occurring at the chlorophyll fraction.

## 2. Materials and Methods

### 2.1. Microorganisms and Culture Media

The axenic culture of *Scenedesmus obliquus* (CPCC05) was supplied by the Canadian Phycological Culture Centre (Waterloo, Canada). We applied the following incubation conditions, 26 °C, photon flux density of 30 µmol m^−2^ s^−1^ and a photoperiod of 12 h to obtain the stock cultures, which were propagated and maintained in synthetic BG11 medium [40].

### 2.2. Cultivation Conditions

The phototrophic experiments were carried out in a 2 L bubble column photobioreactor (Tecnal, Piracicaba-SP, Brazil) operated in batch mode [41]. We applied the following experimental conditions: 100 mg/L for the initial cell concentration, and 26 °C for the isothermal reactor, which was fed with 2 L of B11 medium, pH set to 7.6, 150 µmol m^−2^ s^−1^ for the photon flux density and a light cycle of 24:0 h (light:dark). Continuous aeration of 1 VVM (volume of air per volume of culture per minute) was applied with the injection of air enriched with 15% carbon dioxide. The conditions for the heterotrophic cultivations were set up in a 2 L bubble column bioreactor operating under a batch regime [42]. It was operated at 26 °C in the absence of light, with a carbon/nitrogen ratio of 20, pH adjusted to 7.6, aeration of 1 VVM, and initial cell concentration of 100 mg/L. The culture medium consisted of BG11 synthetic medium supplemented with 12.5 g/L of D-glucose.

### 2.3. Kinetic Parameters

We used the biomass data to calculate the biomass productivity [P_X_ = (X_i_ − X_i−1_) × (t_i_ − t_i−1_)^−1^, mg/L h], the maximum specific growth rate [ln(X_i_/X_0_) = µ_max_ × t, 1/h], and generation time [tg = 0.693/µ_max_, h]. Hence the X_i_ is the biomass concentration at time t_i_ (mg/L), while X_i−1_ is the biomass concentration at time t_i−1_ (mg/L) and X_0_ is the biomass concentration at time 0. μ_max_: maximum specific growth rate (h–1). Residence time (t, in h) is defined as the time required for cells to reach the end of the stationary phase.

### 2.4. Extraction of Photosynthetic Pigments

Aliquots of microalgae or cyanobacteria biomass (5 mL) were filtered with a Whatman grade GF/F glass microfiber filter (47-mm diameter, Merck, Darmstadt, Germany), and immediately frozen at −80 °C [43]. The filter was grinded with liquid nitrogen into powder and mixed with 10 mL of DMF:water (9:1) under stirring at 4 °C for 15 min and spinning (10,000 rpm, 5 min). Subsequently, the solvent phase was collected in a separation funnel whereas the solid residue was re-extracted with 10 mL hexane, ultrasonicated (5 min, 720 W), and vortexed (5 min). Then, 10 mL NaCl solution (10% *w/v*) was added to the mixture, centrifuged (10,000 rpm, 5 min) and the supernatant was added to the first extract in the funnel. Finally, the pellet was dissolved with 10 mL diethyl ether in an ultrasonic bath (5 min, 720 W) and finally vortexed for 5 min. Then, the solution was mixed with 10 mL NaCl solution (10% *w/v*) and the mixture was centrifuged (10,000 rpm, 5 min) and added to the previous extracts in the funnel. There, the mixed solvent layers were extracted with diethyl ether and NaCl solution (10% *w/v*). The water layer was discarded, and the organic phase was concentrated to dryness in a rotary evaporator. The residue was dissolved in acetone. Samples were stored at −20 °C until analysis within 1 week.

### 2.5. Identification of Photosynthetic Pigments by HPLC-ESI/APCI-HRTOF-MS^n^

The chromatographic separation of the individual chlorophyll derivatives and carotenoids was achieved in a Dionex Ultimate 3000RS U-HPLC equipment (Thermo Fisher Scientific, Waltham, MA, USA). The column applied for chlorophyll pigments was a reversed-phase C18 column (200 × 4.6 mm i.d., Teknokroma, Barcelona, Spain), 3 µm particle size, while the elution gradient was the one described previously [44]. The separation of the carotenoid profile required different chromatographic conditions. A reversed-phase C30 column (250 × 4.6 mm i.d., YMC, Schermbeck, Germany), with 3 µm particle size, was applied with the elution gradient described earlier [45,46]. For chlorophyll and carotenoids, the injection volume was 30 µL and the flow rate utilized was 1 mL/min. The UV-visible spectra of the chromatographic peaks were recorded in the 300–700 nm range with a PDA detector. Subsequently, a split post-column of 0.4 mL/min was introduced directly on the mass spectrometer ion source (micrOTOF-QIITM High Resolution Time-of-Flight mass spectrometer with Qq-TOF geometry, Bruker Daltonics, Bremen, Germany). The analysis was developed with an ESI interface (for chlorophyll compounds) or an APCI source (for carotenoid compounds). The instrument was operated in positive ion mode and scanning the *m/z* values in the 50–1200 Da range. We operated the acquisition of the mass spectra in broad-band Collision Induced Dissociation mode (bbCID), so that MS and MS/MS spectra were recorded simultaneously. The instrument control was performed with Bruker Compass HyStar software (Bruker Daltonics version 3.2, Bremen, Germany), whereas the processing of MS data was made with the Bruker Compass DataAnalysis software (Bruker Daltonics version 4.1, Bremen, Germany). For the automated screening of signals corresponding to identified chlorophyll derivatives and carotenoids on the EICs, we applied the TargetAnalysis^TM^ software (Bruker Daltonics version 1.2, Bremen, Germany). The validation of the automated identifications was carried out according to different filtering rules, including mass accuracy (tolerance limit set at 5 ppm) and isotopic pattern comparison calculated with the SigmaFit^TM^ (Bremen, Germany) algorithm (tolerance limit set at 50) [44]. The interpretation of the MS/MS spectra and the consistency of the product ions, which have to fulfil the previous filtering rules for mass accuracy and isotopic pattern, was developed with the SmartFormula3D^TM^ (Bremen, Germany) module [44]. The software MassFrontier^TM^ software (Thermo Scientific^TM^ version 4.0, Waltham, MA, USA) allowed the acquisition of the in silico tandem MS spectra of the filtered analytes to compare the theoretical product ions with the corresponding experimental ones. This software allows the evaluation of different product ions when different isomers show the same bbCID spectrum.

### 2.6. Quantification of Photosynthetic Pigments by HPLC-UV-Visible Detection

The identified pigments were quantified by reversed-phase HPLC using a Hewlett-Packard HP 1100 liquid chromatograph with the same columns and eluent gradients as for the MS analyses. The on-line UV-visible spectra were recorded in the 350–800 nm wavelength range. Sequential detection was performed at 410, 430, 450, and 666 nm with a photodiode-array detector. Data were collected and processed with the HP ChemStation (Rev.A.05.04) software (Agilent Technologies, Waldbronn, Germany). Calibration curves (amount versus integrated peak area) were obtained by the least-squares linear regression analysis for quantification of pigments. The concentration range considered to build the calibration equations was ascertained from the observed levels of the pigments in the samples. Triplicate injections were made for five different volumes of each standard solution.

### 2.7. Statistical Analysis

Normality of data (mean values of three independent measurements) was checked with the Shapiro-Wilk test, and one-way analysis of the variance was performed using the Statistica software (version 6, StatSoft, Inc., 2001, Palo Alto, Santa Clara, CA, USA). Post-hoc comparison for detecting statistic significant differences was made with the Tukey test, setting the significance value a *p* < 0.05.

## 3. Results

### 3.1. Microalgae Growth/Kinetic Parameters

**Figure 3:**
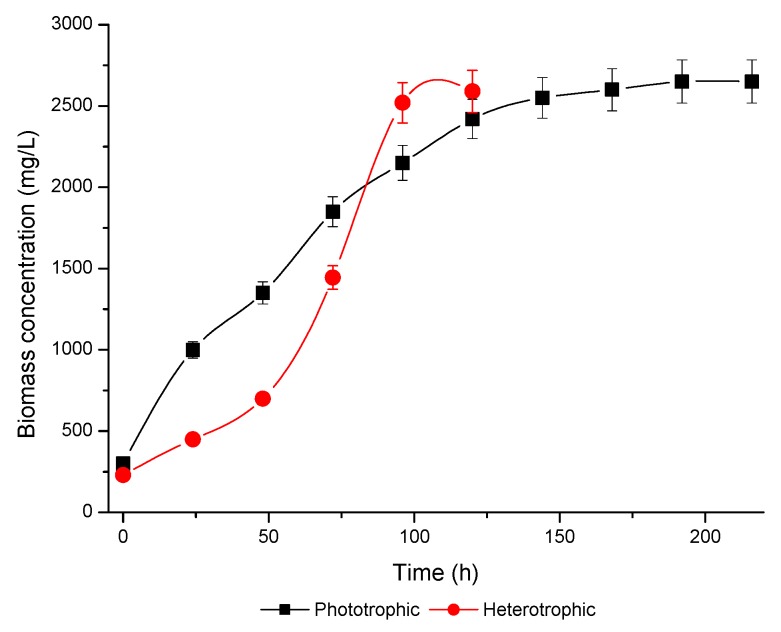
Growth curves in phototrophic and heterotrophic culture regimes of *Scenedesmus obliquus*.

Knowledge regarding the growth pattern of microalgae and its parameters is not only interesting for the quantitative production of both biomass and metabolites, but also for increasing our comprehension of both the synthesis regulation and degradation dynamics of photosynthetic products. In this sense, Figure 3 depicts representative growth curves for the microalgae *Scenedesmus obliquus* through phototrophic and heterotrophic metabolic pathways, whereas the growth parameters are presented in Table 1. Hence, both culture conditions followed an exponential growth from the beginning without lag phase, at the specific growth rates of 0.023 and 0.024 h^−1^, generation intervals of 30.13 and 28.8 h, and finally reaching a stationary phase at 144 h and at 96 h in photosynthetic and heterotrophic cultures, respectively. The highest biomass accumulation was achieved under the photosynthetic cultivation (2650 mg/L) which was only 2% higher than the high biomass concentration of heterotrophic condition (2600 mg/L). The greatest impact of the type of cultivation was on the biomass productivity, where the highest value was obtained under heterotrophy (19.75 mg/L h), which is a consequence of the low residence time (120 h) reached in this condition, when compared with the phototrophic culture (216 h) that resulted in a productivity of 10.87 mg/L h. Regardless, at very long incubation times during the phototrophic growth, it is impossible to ensure that no nutrient deprivation occurs. However, assuming this possibility, we extended the study to analyze the effects of excess of light on pigment composition.

### 3.2. Pigment Profile During Phototrophic Growth

**Figure 4:**
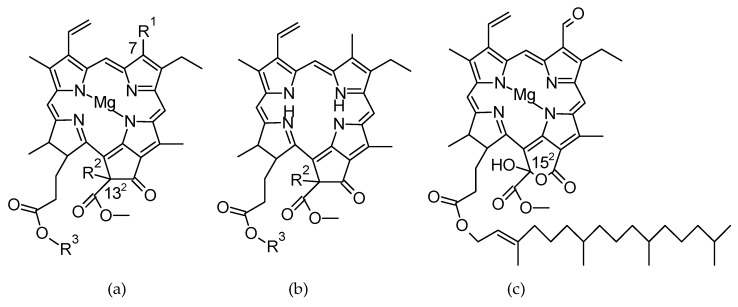
Chlorophyll structures identified in *Scenedesmus obliquus*: (**a**) chlorophyll (R^3^ is phytol, C_20_H_40_) and chlorophyllide (R^3^ is H) structure, R^1^ is CH_3_ for chlorophyll *a*, and CHO for chlorophyll *b*, R^2^ is H for chlorophyll (*a* and *b*) and OH for 13^2^-hydroxy-chlorophyll (*a* and *b*); (**b**) pheophytin (R^3^ is phytol, C_20_H_40_) and pheophorbide (R^3^ is H) structure, R^2^ is H for pheophorbide, and OH for 13^2^-hydroxy-pheophorbide; (**c**) 15^2^-hydroxy-lactone chlorophyll *b* structure.

The characteristics of the chromatographic and mass spectrometric data for the different pigments analyzed in the present study are shown in Table S1. *Scenedesmus* exhibits the typical carotenoid profile of the Chlorophyta taxon, which mainly contains lutein, β-carotene, and relative amounts of minor xanthophylls, such as violaxanthin and neoxanthin [47]. The chlorophyll fraction has been generally described as comprised by chlorophyll *a* and chlorophyll *b*, a feature of this taxonomic group of green algae. However, our detailed analysis reveals the presence of intermediary chlorophyll metabolites within the chlorophyll profile. Figure 4 displays the structures of the chlorophyll derivatives present in the profile of *Scenedesmus obliquus*.

It was observed that the total amount of carotenoids (Table 2) increased with the radiation time until the microalgae reached the stationary phase (144 h), to subsequently present a steady state until the end of the phase. In *Scenedesmus*, this behavior is due to the response of the main carotenoids, lutein and β-carotene, to the continuous illumination. As it has been stated [39], the same carotenoid kind may develop different roles in the cell depending on its location. According to the observed data (Table 2), lutein and β-carotene behave as primary photosynthetic pigments in *Scenedesmus*, although β-carotene could perform secondary activities in other chlorophytes, and even transported into oil droplets where they accumulate under stress conditions [17]. Regardless, lutein and β-carotene are photoprotective pigments, minimizing the photoinhibition through additional roles as quenchers or scavengers [39]. However, the minor xanthophylls display a different behavior under continuous radiation in *Scenedesmus* cells. Neoxanthin, violaxanthin, luteoxanthin, and antheraxanthin increased their concentrations in the microalgae culture even after the stationary growth phase. Specifically, violaxanthin and antheraxanthin are involved in the so-called xanthophyll cycle, intimately related with the ability to dissipate the excess of absorbed light. During high light irradiance conditions, the de-epoxidation reaction of violaxanthin to produce antheraxanthin reduces the light-harvesting efficiency in the antenna [48]. Finally, although neoxanthin could be considered as a light harvesting pigment, it also develops a role as photoprotective compound, reacting towards reactive oxygen species and preventing cell damage [49].

In relation to the response of the chlorophyll fraction to the continuous irradiance (Table 3), it was observed that light exposure initially induces chlorophyll synthesis. Although this result was anticipated, prolonged irradiance times (which means an excess of light) result in a net degradation of the chlorophyll fraction. The detailed analysis of the chlorophyll profile during the phototrophic growth of *Scenedesmus* shows chlorophyll *a* and *b* as the main pigments, but the accumulation of the intermediary metabolites pheophytin and pheophorbide *a* was also concomitant. Pheophytin *a* (Figure 4b) is produced by the substitution of the central Mg^2+^ ion by hydrogens, while pheophorbide *a* (Figure 4a) involves an additional dephytylation step at the C17^3^ position. However, the outstanding results are the production of a heterogeneous profile of oxidized chlorophylls. Among them, the 13^2^-hydroxy-compounds stand out, which result from the oxidation at the C13^2^ carbon atom (R^2^ is OH in Figure 4) in chlorophyll of the *a* and *b* series, and in pheophorbide *a*. Furthermore, the formation of a lactone functional group is considered a further step in the oxidative level of the original chlorophyll structure [31]. In this sense, it was very surprising to find 15^1^-hydroxy-lactone chlorophyll *b* (Figure 4c) in the chlorophyll profile of *Scenedesmus* under radiation conditions.

It is noteworthy to highlight the different behavior of chlorophyll derivatives from *a* series (Figure 4a, CH_3_ at C7) from that observed for the *b* series (Figure 4a, CHO at C7). The chlorophyll compounds from *a* series, except pheophytin *a*, were biosynthesized until the maximum growth stage was reached (144 h), and subsequently a progressive degradation initiated. However, metabolites from chlorophyll *b* series (chlorophyll *b*, 13^2^-hydroxy-chlorophyll *b* and 15^1^-hydroxy-lactone chlorophyll *b*) showed their maximum concentrations between 48 and 72 h of illumination, around half of the period required to reach the residence time. After the apex peak, the metabolites of the chlorophyll *b* initiated a net degradation. The interconversion of chlorophyll *a* and *b*, through the denominated chlorophyll cycle (Figure 1 [50]), is an essential mechanism in photosynthetic organisms, as they can adapt their photosynthetic apparatus to the irradiance level. At high levels of illumination, the organism reduces the antenna complexes to avoid excess of photons, so that the production of reactive oxygen species (ROS) is minimized. As antenna complexes are rich in chlorophyll b compounds, at high irradiances the relative amounts of chlorophyll *b* decreased. On the contrary, at low irradiance (shadow) conditions, the organism rises the antenna complexes to capture as many photons as possible, which results in an increase of the chlorophyll compounds of the *b* series. Consequently, microalgae modify the ratio of chlorophyll *a/b* according to the irradiance levels [51]. As it can be observed in Table 4, at the initial 72 h of growth the ratio of *a/b* series decreased in *Scenedesmus*, as the biosynthesis rate of chlorophyll *b* was higher than that for the chlorophyll *a*. However, when the quantity of light was excessive for the culture (after 72 h of continuous illumination), the antenna complexes decreased, the concentration of chlorophyll *b* diminished and, consequently, the *a/b* ratio increased. Similar changes in the *a/b* ratio have been observed for *Chlorella* and *Dunalliela* [52]. At high irradiances, the energy received by chlorophyll *a* molecule is higher than its capacity to transfer it towards the photosynthetic electron transport chain, and chlorophyll *a* switches to the triplet excited stage [39]. Next, overexcited chlorophyll *a* molecule is quenched by molecular oxygen yielding ROS. As we can observe in Table 3 and Table 4, the interconversion between chlorophyll *a* and *b* contents is the preferred mechanism of *Scenedesmus* cells to avoid the formation of ROS at high irradiances.

Nevertheless, once the maximum concentrations for chlorophyll *a* (144 h, Table 3) and *b* (72 h in Table 4) were reached, the net degradation of chlorophyll compounds was not exhaustive. Otherwise, *Scenedesmus* cells reached a steady state for the chlorophyll content (around 6200 mg/kg dw. for chlorophyll *a*, 1900 mg/kg dw. for chlorophyll *b*) until the end of the controlled period. It seems that once the top biosynthetic capabilities were accomplished, the microalgae found an ‘ideal’ chlorophyll content, which allows an equilibrated photosynthetic performance, that is, a productive one but not harmful, at least at the irradiance assayed for *Scenedesmus*. As it has been previously stated, photoacclimation is complete only when a balanced growth condition is achieved [53]. However, this is accurate when the chlorophyll content is determined as a whole value. As we have shown, a detailed study of the complete chlorophyll profile allows to observe different biosynthetic capabilities with some chlorophyll metabolites reaching steady state earlier, precisely to fit with the photoacclimation at 144 h.

Pheophorbide *a* and pheophytin *a* are currently considered the metabolites of the chlorophyll degradation pathway (Figure 1). In fact, pheophorbide, pheophytin, and pyropheophorbide have been associated with the chlorophyll degradation in cyanobacteria in sedimentary surfaces [54] and chlorophyll senescence in marine environments [34]. Recently, the gene responsible of the formation of pheophytin (SGR) a has been identified in *Chlamidomonas reinharditii* [24]. However, while the kinetics of production and degradation of pheophorbide *a* is parallel to the chlorophyll *a*, the profile of metabolism of pheophytin *a* seems to progress in a different fashion and not correlated with the metabolism of chlorophyll *a*. The maximum concentration of pheophytin *a* was observed at 24 h, while its progressive decay through the continuous illumination period made the interpretation of the results in base to its implication in the chlorophyll degradation pathway challenging.

In addition, the HPLC-ESI/APCI-hrTOF-MS analyses of the chlorophyll fraction revealed the existence of a specific chlorophyll oxidative metabolism (Table 2 and Table 3) during the *Scenedesmus* phototrophic cultivation. As stated before (Figure 2), hydroxylation at C13^2^ is the first step in the oxidative pathway of chlorophylls. Hence, 13^2^-hydroxy-chlorophyll *a* and *b* increased their concentrations in the cell with the continuous illumination for the initial 72 h period, and afterwards a progressive degradation was observed. In any case, it is important to highlight that the maximum of 13^2^-hydroxy-chlorophyll *a* cellular content was not concurrent with the maximum concentration of chlorophyll *a*, which pointed towards a specific linking reaction between both compounds instead of an unspecific process. Noteworthy, 13^2^-hydroxy-pheophorbide *a* was also produced around 3 days of illumination, once pheophorbide *a* is biosynthesized in the microalgae. A further oxidative process is the generation of the lactone rearrangement at the C15^1^ position (Figure 4). During the phototrophic growth of *Scenedesmus* a progressive accumulation of 15^1^-hydroxy-lactone chlorophyll *b* is observed, reaching the maximum value after 72 h (Table 3). In our experimental conditions it seems 72 h is the timeframe for *Scenedesmus* to reach the ‘buffer capacity’ (from the point of view of chlorophylls) and manage both the excess of energy and, consequently, the potential accumulation of ROS. Afterwards, profound physiological changes are required to avoid oxidative stress, as the commented restructuration of antenna complexes.

Moreover, no 15^1^-hydroxy-lactone chlorophyll *a* formation was detected in any moment of the phototrophic growth, although chlorophyll *a* is the main chlorophyll pigment in the chlorophyll profile of *Scenedesmus*. In fact, although a chlorophyll metabolite with this functional group is not easy to distinguish [1], it is the 15^1^-hydroxy-lactone chlorophyll *a* catabolite observed (if any) in photosynthetic organisms, but not the 15^1^-hydroxy-lactone chlorophyll *b* catabolic product [30]. Indeed, both proportionally and in absolute concentration, the total biosynthesized chlorophyll oxidative compounds of the *b* series overcame those of the *a* series. To the best of our knowledge, this is the first time to describe such phenomenon. The biochemical origin of the oxidized chlorophyll metabolites is still under discussion. In higher plants, different enzymatic systems have been assumed as responsible for such oxidation (lipoxygenase and/or peroxidase) [29,30,31]. However, although different oxidative mechanisms have been observed in microalgae (peroxidase, superoxide dismutase, polyphenol oxidase, glutathione peroxidase, etc.) [55,56], none of them have been correlated with the chlorophyll metabolism so far. Two possible hypotheses can explain the higher rate of oxidation of chlorophyll *b* catabolism. Thus, the preferential accumulation of chlorophyll *b* catabolites could be due to an unknown chlorophyll *b* affinity by the pool of oxidative enzymes pool, or this singularity could be caused by the different localization of both chlorophyll series in the photosynthetic apparatus. Further research is required to unravel the exact mechanism.

### 3.3. Pigment Evolution During Heterotrophic Growth

As it can be seen in Table 5, heterotrophy means carotenoid degradation for *Scenedesmus obliquus* in our experimental conditions, although at very different rates depending on the carotenoid sort. The initial 24 h in darkness produces a significant carotenoid degradation except for neoxanthin, while the concentration of β-carotene and violaxanthin decreased by half. This decrease was extended in a lower degree for lutein. From 24 to 48 h of growth in the darkness, carotenoids were highly stable, the next 24 h interlude (72 h) only being a significant stage for the stability of neoxanthin and violaxanthin. Extending the heterotrophic culture of *Scenedesmus obliquus* far from 96 h implied a carotenoid degradation of at least 85%. In fact, carotenoid production in heterotrophic cultivation requires additional oxidative stress: high salt concentration, high light, etc. [13]. In any case, it is important to highlight the different stability of carotenoids in heterotrophic conditions, to face the future biotechnological strategies aimed to enhance the production of carotenoids.

On the contrary, it was remarkable to observe the behavior of the chlorophyll fraction at heterotrophic culture conditions. During the initial 48 h of growth, the total amount of chlorophylls was constant and after that time interval, the chlorophyll profile initiated a phase of net degradation with increased rate at the end of the controlled period. Such modification in the chlorophyll metabolism is coincident with an increase of biomass. The initial steady state of the chlorophyll content means that the biosynthetic and the degradative reactions are evolving at the same rate. Although the exact quantity is unknown, the half-life of a chlorophyll molecule is estimated around several hours [57]. This fact implies that during the steady state of chlorophylls in the initial 48 h of heterotrophic culture, biosynthetic and degradative reactions are running in *Scenedesmus* cells. Regarding the biosynthetic metabolism, as stated before green algae can synthesize chlorophylls in dark conditions. Consequently, during 48 h of heterotrophic cultivation of *Scenedesmus*, a continuous synthesis of chlorophylls took place, although at the same rate as the degradative reactions. The first assumption to consider is that under heterotrophic conditions, the cell does not invest energy in chlorophyll synthesis but focuses on the cell division and growth process with the available resources. In fact, it has been argued that glucose can inhibit the chlorophyll biosynthesis, by means of an inhibitory activity towards the precursor coprophorphyrin III [58]. On the contrary, some reports have shown a certain degree of chlorophyll retention during heterotrophic growth [59], as we have found for *Scenedesmus*. The exact physiological meaning of such energetic investment is unknown to date, although our results are an important starting point for future biotechnological applications aimed to enhance the chlorophyll production.

In addition, the detailed analysis of the chlorophyll profile during the heterotrophic growth of *Scenedesmus* shows accumulation of chlorophyll metabolites produced during the chlorophyll degradation, that mirror the masked reactions that were under progress. Pheophorbide, chlorophyllide, and pheophytin are intermediary catabolites during the chlorophyll degradation pathway. Table 5 shows a significant increment of pheophorbide and chlorophyllide *a* at the end of the controlled period, concomitant with the main degradation of chlorophylls. However, pheophytin levels continuously decreased through the cycle, showing no parallelism with the chlorophyll breakdown. The results suggest that the operating pathways during the heterotrophic cultivation of *Scenedesmus* are better related with the chlorophyllase (CHL) pathway (Figure 1) than with pheophytinase one (PPH). Homologous PPH proteins have been found through BLASTP (Basic Local Alignment Search Tool for Proteins) searches in green algae but not in cyanobacteria, and it has been proposed that PPHs are also likely to be operative in the green algae [60], although no functional analysis has been developed so far. Although such data are not available, PPH seems to not be responsible for the chlorophyll degradation during heterotrophic conditions, at least during the culture conditions assayed in *Scenedesmus*.

To the best of our knowledge, accumulation of 13^2^-hydroxy-chlorophylls is described for the first time in this study during the heterotrophic culture of green microalga, although no 15^1^-hydroxy-lactone derivatives were detected. Interestingly, the heterotrophic strategy only induced oxidation in chlorophyll *a* molecules and no oxidized chlorophyll *b* compounds were detected in any moment of the cycle. 13^2^-hydroxy-chlorophyll *a* production, observed during the initial 48 h of growth in the darkness could involve a role during the chlorophyll turnover, although the main synthesis is accomplished with the net degradation of chlorophylls at the end of the cultivation period. As stated before, the exact role of oxidized chlorophylls in phytoplankton is unclear, but associated with defense, grazing, senescence, or even death cell [33,34,35,36,37,38]. Our results show both production and degradation kinetics during the heterotrophic culture of *Scenedesmus*, with more than a plausible role during the chlorophyll degradation. Consequently, the results obtained in Table 5 open a door for future research, with a focus on the biochemical mechanisms involved in the chlorophyll oxidative metabolism during the heterotrophic cultivation of green microalgae.

## 4. Conclusions

As stated in the introduction, the improvement of pigment production with biotechnological parameters requires a deep understanding of the reactions that take place during the different culture approaches. In this sense, it is essential to know the physiological strategies that green microalgae develop to become acclimatized to the environmental conditions. In addition to the technological data, our study introduces a specific and different chlorophyll oxidative metabolism during phototrophic and heterotrophic cultivation, which agrees with the measurement of oxidized chlorophyll metabolites in natural phytoplankton environment [35,36]. Future assays in controlled bioreactors are required to unravel the precise implication of such oxidative metabolism.

## Figures and Tables

**Table 1:** Kinects parameters of *Scenedesmus obliquus* in phototrophic and heterotrophic growth conditions (mean ± SD).

Parameter	Phototrophic	Heterotrophic
X_max_ (mg/L)	2650 ± 111.8	2600 ± 97.5
µ_max_ (h^−1^)	0.023 ± 0.00	0.024 ± 0.00
RT (h)	216 ± 0.00	120 ± 0.00
GT (h)	30.13 ± 0.60	28.8 ± 0.49
P_X_ (mg/L h)	10.87 ± 0.43	19.75 ± 0.29

X_max_: maximum cell biomass; μ_max_: maximum specific growth rate (h^−1^); RT: residence time (h); GT: generation time (h); P_X_: average biomass productivity (mg/L h).

**Table 2:** Evolution of the carotenoid profile during the phototrophic growth of *Scenedesmus obliquus* (mg/kg dw).

Time (h)	Neox	Violax	Luteox	Antherax	Lutein	β-Carotene	Total
0	+	+	+	0.0	703.3	30.0	703.3
24	127.0	11.0	20.0	0.0	689.5	30.1	877.2
48	142.0	10.4	26.3	0.0	860.7	38.9	1174.3
72	130.3	28.1	37.0	7.0	795.4	45.9	1044.0
96	83.7	25.8	40.7	15.6	931.2	53.2	1150.1
120	146.7	28.5	36.7	20.4	1238.3	176.4	1647.0
144	147.0	40.0	45.1	32.0	1443.9	224.7	1952.4
168	122.3	19.8	51.3	26.2	1125.0	220.0	1679.6
192	156.2	38.9	67.0	30.0	1313.2	227.1	1832.4
216	321.9	108.9	44.0	49.2	1408.7	212.6	2145.3

Neox: neoxanthin, Violax: violaxanthin, Luteox: luteoxanthin, Anterax: anteraxanthin, Total: total carotenoids. +, means presence but under the LOQ. (coefficient of variance < 10% in all cases).

**Table 3:** Evolution of the chlorophyll profile from series *a* during the phototrophic growth of *Scenedesmus obliquus* (mg/kg dw).

Time (h)	Pheo *a*	OH-Pheo *a*	OH-Chl *a*	Chl *a*	Phy *a*
0	0.0	0.0	0.0	2438.3	2787.7
24	0.0	0.0	85.0	2420.0	3350.0
48	20.4	0.0	111.1	3955.2	2950.7
72	33.0	0.0	148.6	3718.6	2375.9
96	155.6	0.0	114.0	4079.3	2413.7
120	210.0	22.9	133.3	3981.3	2590.0
144	262.7	13.7	127.5	7417.5	897.8
168	86.7	8.3	134.6	5935.8	878.5
192	31.7	11.3	0.0	6170.0	708.7
216	8.5	0.0	0.0	6878.1	192.1

Pheo: pheophorbide, OH-Pheo *a*: 13^2^-hydroxy-chlorophyll *a*, Chl: chlorophyll, OH-Chl *a*: 13^2^-hydroxy-chlorophyll *a*, Phy: pheophytin. (CV < 10% in all cases).

**Table 4:** Evolution of the chlorophyll profile from series *b* during the phototrophic growth of *Scenedesmus obliquus* (mg/kg dw).

Time (h)	OH-Lact.-Chl *b*	OH-Chl *b*	Chl *b*	Series *a/b*
0	100.0	116.7	1408.3	3.22
24	175.0	124.5	1472.5	3.30
48	179.3	418.1	2023.0	2.71
72	275.1	437.6	5557.0	1.02
96	122.6	308.6	1757.2	3.02
120	116.7	310.4	1724.0	3.13
144	132.0	231.8	2016.7	3.55
168	90.0	286.3	1802.7	3.19
192	56.8	31.1	1878.3	3.50
216	40.9	50.6	1747.2	3.85
240	51.0	63.5	2038.6	3.14

OH-Lact.-Chl *b*: 15^1^-hydroxy-chlorophyll *b*; OH-Chl *b*: 13^2^-hydroxy-chlorophyll *b*; Chl *b*: chlorophyll *b*. (CV < 10% in all cases).

**Table 5:** Evolution of the chlorophyll and carotenoid profile during the heterotrophic growth of *Scenedesmus obliquus* (mg/kg dw).

Pigment	Residence Time (h)					
	0	24	48	72	96	120
Neoxanthin	86.4	76.9	80.0	43.5	36.5	13.3
Violaxanthin	42.2	18.7	18.4	10.4	8.5	1.8
Lutein	429.3	323.2	318.3	319.2	284.3	63.2
β-carotene	241.5	144.3	151.2	131.8	128.8	6.6
Chld *a*	3.4	3.5	3.5	3.5	8.5	108.0
Pheo *a*	78.3	157.7	145.0	67.2	37.2	299.9
Chl *b*	2284.0	2644.4	2778.2	1992.9	1164.0	895.4
OH-chl *a*	15.1	111.6	187.5	3.5	3.5	202.5
Chl *a*	4407.1	4343.0	4190.2	4636.3	2841.9	825.4
Phy *a*	1063.5	1102.6	766.1	326.4	262.0	207.0
Tot. carot	799.4	563.2	568.0	505.1	458.2	85.0
Tot. chls	7851.6	8362.9	8070.7	7030.1	4317.4	2538.3

Chld *a*: chlorophyllide *a*; Pheo *a*: pheophorbide *a*; Chl *a*, Chl *b*: chlorophyll *a*, chlorophyll *b*; OH-chl *a*: 13^2^-hydroxy-chlorophyll *a*; Phy *a*: pheophytin *a*; carot: carotenoids. (CV < 10% in all cases).

## Supplementary Materials

The following are available online at https://www.mdpi.com/2076-3921/8/12/600/s1, Table S1: Photosynthetic pigments identified by HPLC-PDA-ESI/APCI(+)-Q-TOF in the study.
