# Supplementary material for: Chlorophyll Oxidative Metabolism During the Phototrophic and Heterotrophic Growth of Scenedesmus obliquus
Source: Antioxidants (Basel). 2019 Nov 29;8(12):600. doi: 10.3390/antiox8120600 (PMC6943719; doi:10.3390/antiox8120600)
Supplement: Supplementary file 1 [file antioxidants-08-00600-s001.pdf]

Table S1. Photosynthetic pigments identified by HPLC-PDA-ESI/APCI(+)-Q-TOF in the study.

Pigment	UV-Vis Maxima (nm)	$[M + H]^+$ (*m*/*z*)	Elemental Composition
chlorophyllide *a*	430, 666	615.2450	$C_{35}H_{34}MgN_4O_5$
pheophorbide *a*	410, 665	593.2761	$C_{35}H_{36}N_4O_5$
pheophorbide *a´*	409, 666	593.2758	$C_{35}H_{36}N_4O_5$
13^2-hydroxy-pheophorbide *a*	408, 666	609.2705	$C_{35}H_{36}N_4O_6$
cis-violaxanthin	412, 434, 463	599.4116[a]	$C_{40}H_{56}O_4$
cis-neoxanthin	412, 434, 462	599.4113[a]	$C_{40}H_{56}O_4$
cis-luteoxanthin	398, 421, 448	599.4114[a]	$C_{40}H_{56}O_4$
all-trans-lutein	421, 444, 474	551.4245[b]	$C_{40}H_{56}O_2$
cis-lutein	342, 421, 444, 474	551.4248[b]	$C_{40}H_{56}O_2$
15^2-hydroxy-lactone-chlorophyll *b*	454, 634	939.5115	$C_{55}H_{70}MgN_4O_8$
13^2-hydroxy-chlorophyll *b*	462, 650	923.5169	$C_{55}H_{70}MgN_4O_7$
chlorophyll *b*	466, 650	907.5222	$C_{55}H_{70}MgN_4O_6$
chlorophyll *b´*	466, 650	907.5220	$C_{55}H_{70}MgN_4O_6$
13^2-hydroxy-chlorophyll *b´*	462, 650	923.5164	$C_{55}H_{70}MgN_4O_7$
13^2-hydroxy-chlorophyll *a*	432, 666	909.5380	$C_{55}H_{72}MgN_4O_6$
chlorophyll *a*	432, 666	893.5429	$C_{55}H_{72}MgN_4O_5$
chlorophyll *a´*	432, 666	893.5425	$C_{55}H_{72}MgN_4O_5$
pheophytin *a*	407, 665	871.5730	$C_{55}H_{74}N_4O_5$
pheophytin *a´*	407, 665	871.5731	$C_{55}H_{74}N_4O_5$
all-trans-β-carotene	424, 451, 477	537.4435	$C_{40}H_{56}$
9-*cis*-β-carotene	340, 420, 446, 472	537.4445	$C_{40}H_{56}$

[a]Protonated ion arising from the $M^{\cdot +}$ radical ion. [b]Product ion corresponding to the loss of water from the protonated molecular ion.
